# Cytoplasmic Escape of Mitochondrial DNA Mediated by Mfn2 Downregulation Promotes Microglial Activation via cGas‐Sting Axis in Spinal Cord Injury

**DOI:** 10.1002/advs.202305442

**Published:** 2023-11-27

**Authors:** Fei‐Long Wei, Tian‐Fu Wang, Chao‐Li Wang, Zhen‐Peng Zhang, Jing‐Wei Zhao, Wei Heng, Zhen Tang, Ming‐Rui Du, Xiao‐Dong Yan, Xiao‐Xiang Li, Zheng Guo, Ji‐Xian Qian, Cheng‐Pei Zhou

**Affiliations:** ^1^ Department of Orthopaedics Tangdu Hospital Fourth Military Medical University Xi'an 710038 China; ^2^ Department of Pharmaceutical Analysis School of Pharmacy Fourth Military Medical University Xi'an 710032 China; ^3^ State Key Laboratory of Proteomics Beijing Proteome Research Center National Center for Protein Sciences Beijing Research Unit of Proteomics & Research and Development of New Drug of Chinese Academy of Medical Sciences Institute of Lifeomics Beijing 102206 China

**Keywords:** biomimetic nanoparticles, mitofusin 2, mtDNA, neuroinflammation, spinal cord injury, stimulator of interferon genes (Sting)

## Abstract

Neuroinflammation is associated with poor outcomes in patients with spinal cord injury (SCI). Recent studies have demonstrated that stimulator of interferon genes (Sting) plays a key role in inflammatory diseases. However, the role of Sting in SCI remains unclear. In the present study, it is found that increased Sting expression is mainly derived from activated microglia after SCI. Interestingly, knockout of Sting in microglia can improve the recovery of neurological function after SCI. Microglial Sting knockout restrains the polarization of microglia toward the M1 phenotype and alleviates neuronal death. Furthermore, it is found that the downregulation of mitofusin 2 (Mfn2) expression in microglial cells leads to an imbalance in mitochondrial fusion and division, inducing the release of mitochondrial DNA (mtDNA), which mediates the activation of the cGas‐Sting signaling pathway and aggravates inflammatory response damage after SCI. A biomimetic microglial nanoparticle strategy to deliver MASM7 (named MSNs‐MASM7@MI) is established. In vitro, MSNs‐MASM7@MI showed no biological toxicity and effectively delivered MASM7. In vivo, MSNs‐MASM7@MI improves nerve function after SCI. The study provides evidence that cGas‐Sting signaling senses Mfn2‐dependent mtDNA release and that its activation may play a key role in SCI. These findings provide new perspectives and potential therapeutic targets for SCI treatment.

## Graphical abstract

Downregulation of mitofusin (Mfn2) expression in microglial cells induces the release of mtDNA, which in turn mediates the activation of stimulator of interferon genes (Sting) and aggravates the inflammatory response damage after spinal cord injury (SCI). A biomimetic nano‐delivery platform alleviates activation of the cGas‐Sting pathway by delivering Mfn2 agonists to mitochondria, thereby rescuing neuroinflammation and SCI outcomes.

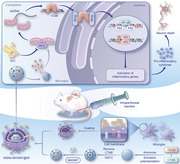

## 1 Introduction

Spinal cord injury (SCI) is the most severe complication of spinal trauma, causing loss of sensory and motor functions below the level of injury.^[^ ^1^ ^]^ Loss of independence and increased lifetime mortality rates are the hallmark effects of SCI.^[^ ^1^ ^]^ Lifetime direct care costs per patient with SCI ranged from $1.1 to $4.6 million.^[^ ^1^ ^]^ Because SCI cannot be prevented, the development of effective treatments is critical. The primary injury period often occurs during acute crush contusions, laceration injuries, shear injuries, and other injuries, followed by a secondary injury period during which ischemia and hypoxia occur in the extraneuronal environment.^[^ ^2^ ^]^ Therefore, treatment of SCI focuses on blocking the generation and development of this damage cascade amplification effect. However, the underlying mechanisms remain unclear.

Microglia are innate immune cells of the central nervous system (CNS) that play a key role in the regulation of neuroinflammatory processes.^[^ ^3^ ^]^ They have dual effects on neuroinflammation and neurogenesis depending on their polarization.^[^ ^4^ ^]^ The classic M1 phenotype of macrophages secretes pro‐inflammatory cytokines, such as tumor necrosis factor (TNF)‐α and interleukin (IL)−1β, which can negatively affect neurogenesis. Microglial activation and neuroinflammation play key roles in secondary injury.^[^ ^5^ ^]^ Therefore, regulating activated microglia opens new avenues for the treatment of SCI.

In recent years, research on activating the stimulator of interferon genes (Sting, Sting1, or Tmem173) signaling pathway has gained significant momentum.^[^ ^6^ ^]^ Sting is an innate immune adapter protein that cooperates with cyclic GMP‐AMP synthase (cGas) to initiate interferon‐based responses that protect the host.^[^ ^7^ ^]^ cGas is a nuclear and cytoplasmic protein that responds to cytoplasmic double‐stranded DNA (dsDNA) molecules by catalyzing the formation of cyclic GMP‐AMP (cGAMP), a second messenger that initiates inflammatory responses via Sting.^[^ ^8^ ^]^ There is an increasing consensus that self‐DNA promotes the activation of Sting, similar to pathogenic DNA.^[^ ^9^ ^]^ Self‐DNA,^[^ ^10^ ^]^ which includes nuclear DNA (nDNA) fragments and mitochondrial DNA (mtDNA), leaks into the cytoplasm or extracellular space following DNA damage. Leakage of mtDNA after tissue injury activates Sting signaling.^[^ ^11^ ^]^ Liao et al.^[^ ^12^ ^]^ found that the histone deacetylase 3 (HDAC3)‐p65‐cGas‐Sting pathway is key to neuroinflammation induced by ischemic stroke. In addition, Sting can regulate the polarization of microglia, and thereby regulate inflammation and neuroinflammation after ischemic stroke.^[^ ^11b^ ^]^ Cellular stress can disrupt the balance within mitochondria, leading to the opening of the mitochondrial permeability transition pore (mPTP) and the release of mtDNA into the cytoplasm. mtDNA can activate inflammatory responses through the cGas‐Sting signaling axis.^[^ ^13^ ^]^ However, whether SCI leads to mtDNA release, Sting signal activation, and proinflammatory responses remains unknown.

Accordingly, we investigated the effects of cGas‐Sting signaling activation in microglia on SCI and explored the underlying mechanisms. By combining the results of bulk‐RNA sequencing (bulk RNA‐seq) and single‐cell mRNA sequencing (scRNA‐seq), we found that Sting was activated after SCI and triggered an inflammatory response. Using C‐X3‐C motif chemokine receptor 1 (Cx3cr1)‐Cre ER^T2^/Sting^fl/fl^ mice as an animal model, we demonstrated that Sting plays a key role in SCI. In addition, we found that the downregulation of mitofusin 2 (Mfn2) expression in microglial cells led to an imbalance of mitochondrial fusion and division, inducing the release of mtDNA, which in turn mediated the activation of the cGas‐Sting signaling pathway and aggravated the inflammatory response damage after SCI. Nanomaterials developed on this basis may have protective effects and are expected to provide a potential therapeutic approach for SCI.

## 2 Results

### 2.1 The cGas‐Sting Pathway Was Activated in SCI Mice

**Figure 1:**
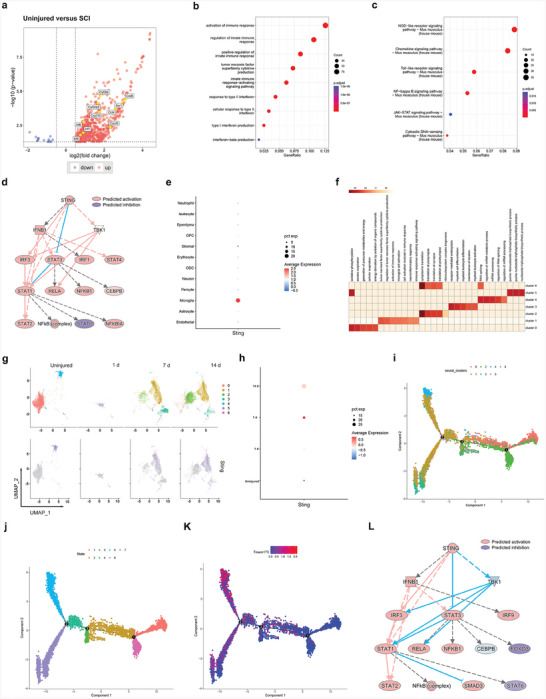
The cGas‐Sting pathway is activated in SCI mice. a) Volcano plot of RNA‐seq data from spine tissue from 8‐week‐old C57BL/6 uninjured and SCI mice. Red and blue dots represent genes with a log2 FC (fold change) of >0.5 and < −0.5, respectively. All other genes are colored gray. Selected genes such as Irf, Cxcl, and Ccl are labeled. b) The GO enrichment analysis revealed hallmark pathways associated with the DEGs, which are upregulated in the control samples compared to the SCI samples. c) KEGG enrichment analysis revealed hallmark pathways associated with the DEGs, which are upregulated in control samples compared to SCI samples. d) IPA prediction of Sting (Sting1, Tmem173) as an upstream regulator of upregulated DEGs identified using an activation z score of >1 and a *p*‐value overlap of <0.05. e) Dot plot of normalized cell‐type expression of Sting (Sting1, Tmem173) in snRNA‐seq samples (*n* = 1); OPCs, oligodendrocyte progenitor cells; ODC, oligodendrocytes. f) The GO enrichment analysis revealed hallmark pathways associated with spinal cord microglia clusters (subtypes). g) UMAP plots showing temporal changes of spinal cord microglia clusters (subtypes) and indicating temporal changes of Sting in microglia. h) Dot plot indicating temporal expression changes of Sting (Sting1, Tmem173) in spinal cord microglia clusters (subtypes). i,k) Single‐cell trajectory and pseudo‐time analysis of microglia clusters defined the proliferation advantage cluster and the metabolism advantage one. l) IPA prediction of Sting (Sting1, Tmem173) as an upstream regulator of upregulated DEGs identified using an activation z score of >1 and a *p*‐value overlap of < 0.05.

Bulk RNA‐seq data from uninjured mice and mice with SCI were analyzed to characterize the changes in gene expression in SCI. Sting was upregulated in mice with SCI compared to uninjured mice, along with significantly upregulated immune response, TNF, and interferon (IFN)‐sensitive response elements (**Figure** **1**a–c). Ingenuity Pathway Analysis (IPA) was used to predict regulators of upregulated differentially expressed genes (DEGs), which confirmed components of IFN, nuclear factor kappa B (NF‐κB), and Janus kinase‐signal transducer and activator of transcription (Jak‐Stat) signaling (IFNB1, STAT3, STAT1, NF‐κB1, and interferon regulatory factor 3 [IRF3]). The cGas‐Sting pathway (STING and TANK‐binding kinase 1 [TBK1]) was predicted to activate the upregulated DEGs (Figure 1d). In the scRNA‐seq data, Sting was primarily detected in microglial clusters (Figure 1e). Microglia is the most abundant cell type found in the spinal cord following SCI.^[^ ^14^ ^]^ A total of seven microglial subpopulation clusters were identified (Figure S1a, Supporting Information). Cluster 1 is associated with inflammatory responses (Figure 1f). One day after the injury, cluster 5 cells were the main microglial subtype detected. With increasing injury time, cluster 1 became the predominant homogeneous microglial subtype. Interestingly, Sting was mainly expressed in cluster 1 (Figure 1g). In addition, a dot plot showed that Sting exhibited the highest expression 7 d after SCI (Figure 1h). Importantly, trajectory and pseudo‐time analyses via Monocle showed that the cells mainly transformed from uninjured cluster 0 to other clusters, and finally transformed into cluster 1 (Figure 1i–k). We then analyzed the DEGs in these two clusters (cluster 0 and cluster 1). Sting was upregulated in cluster 1 compared to cluster 0, and the immune response, TNF, and INF‐sensitive response elements were enriched (Figure S1b,c, Supporting Information). IPA was used to predict the regulators of the upregulated DEGs, which confirmed the components of IFN, NF‐κB, and Jak‐Stat signaling (STAT3, STAT1, NF‐κB, and IRF3). The cGas–Sting pathway (STING and TBK1) was predicted to activate the upregulated DEGs (Figure 1l). Overall, the above findings indicate the activation of the cGas‐Sting pathway after SCI and its association with neuroinflammation.

### 2.2 Activation of the cGas‐Sting Pathway Played a Key Role in Secondary SCI

**Figure 2:**
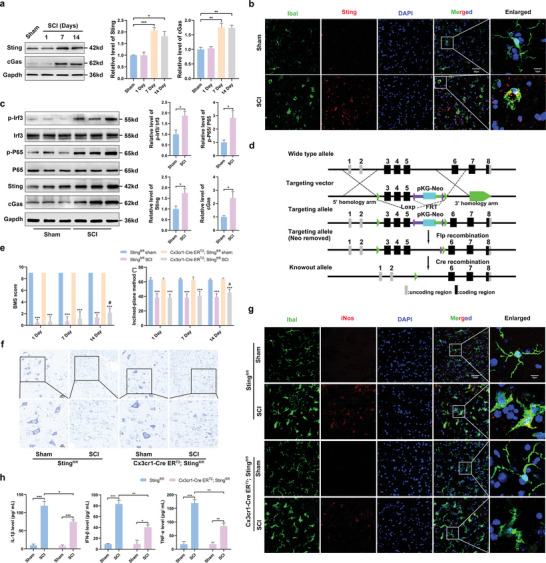
Activation of cGas‐Sting signal played a key role in the secondary SCI. a) Western blots analysis of cGas and Sting levels in the perilesional tissues at 1, 7, and 14 d after SCI. b) Sting /Iba1 double immunostaining in the perilesional tissues 7 d after SCI (Scale bar: 50 µm). c) Western blot analysis of cGas, Sting, Irf3, p‐Irf3, P65, and p‐P65 protein expression in the perilesional tissues 7 d after SCI. d) Diagram for construction of microglia‐specific Sting knockout mice. e) Effects of Sting knockout on neurological function scores at 1 d, 7 d, and 14 d after SCI. f) Representative confocal images of M1 state (iNOS^+^/Iba1^+^) were obtained from the perilesional tissues 7 d after SCI. g) Nissl staining in the perilesional tissues 7 d after SCI (Scale bar = 20 µm). h) Levels of pro‐inflammatory cytokines, including IL‐1β, IFN‐β, and TNF‐α in the perilesional tissues 7 d after SCI. *n* = 6 for each group. Error bars denote mean ± SEM, ns, no significance, ^***^ *p* < 0.001 versus sham group in each strain of mice, # *p* < 0.05 versus Cx3cr1‐Cre ERT2; Stingfl/fl group.

The expression of Sting and cGas proteins increased significantly with time after SCI and peaked on day 7 (**Figure** **2**a). Using double immunostaining of Sting with a cell marker (ionized calcium‐binding adaptor molecule 1, Iba1), we further confirmed the increased expression of Sting in activated microglia of mice with SCI (Figure 2b). It has been reported that activation of Sting can promote the phosphorylation of Irf3 and P65.^[^ ^11a^ ^]^ In the present study, we consistently found that Irf3 and p65 phosphorylation levels significantly increased in mice with SCI (Figure 2c). To better understand the role of Sting in mice with secondary SCI, we created microglia‐specific Sting knockout (MKO) mice using the Cre‐LoxP system (Figure 2d). In MKO mice, Sting expression was significantly reduced in microglia. Neurological function was assessed on days 1, 7, and 14 after SCI. We found that Cx3cr1‐Cre ER^T2^/Sting^fl/fl^ mice had higher Basso Mouse Scale (BMS) and inclined‐plane method scores than Sting^fl/fl^ mice at 14 d after SCI (Figure 2e). It is well established that the quantity and shape of Nissl bodies reflect the degree of metabolic activity and the functional state of neurons.^[^ ^15^ ^]^ The number of Nissl‐positive cells in Cx3cr1‐Cre ER^T2^/Sting^fl/fl^ mice was significantly higher than that in Sting^fl/fl^ mice (Figure 2f; Figure S2a, Supporting Information). The expression of inducible nitric oxide synthase (iNOS) was significantly reduced in Cx3cr1‐Cre ER^T2^/Sting^fl/fl^ mice 7 d after SCI (Figure 2g; Figure S2b, Supporting Information). In addition, pro‐inflammatory cytokines (IFN‐β, TNF‐α, and IL‐1β) were significantly reduced (Figure 2h). In conclusion, the activation of the cGas‐Sting pathway plays a key role in secondary SCI.

### 2.3 The cGas–Sting Pathway Was Activated in Lipopolysaccharide (LPS)‐Induced Microglia

**Figure 3:**
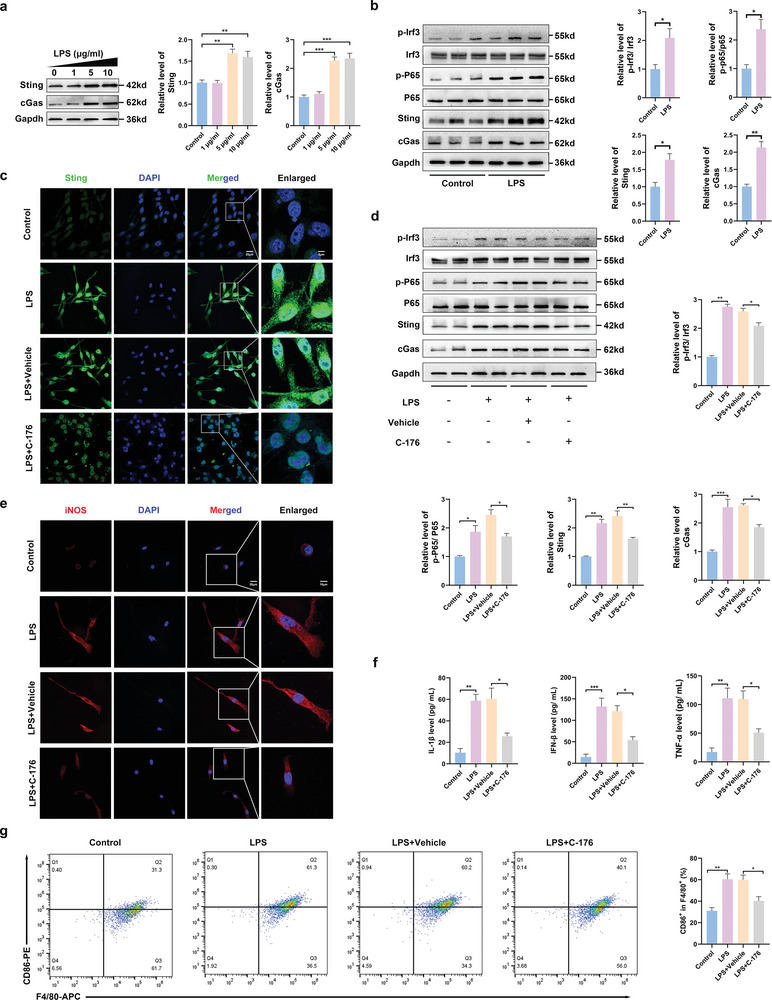
cGas‐Sting signaling pathway was activated in LPS‐induced microglia. a) Western blot and quantitative analysis of cGas and Sting in LPS‐induced microglia of 0, 1, 5, and 10 µg mL^−1^ after 24 h. b) Western blot analysis of cGas, Sting, Irf3, p‐Irf3, P65, and p‐P65 protein expression in LPS (5 µg mL^−1^)‐induced microglia. c) Representative of immunofluorescence staining of Sting in microglia (Scale bar: 20 µm). d) Western blot and quantitative analysis of cGas, Sting, Irf3, p‐Irf3, P65, and p‐P65 in microglia of Control, LPS, LPS + vehicle, and LPS + C‐176. e) Representative immunostained images of M1 state (iNOS) microglia. f) Levels of pro‐inflammatory cytokines, including IL‐1β, IFN‐β, and TNF‐α in the microglia medium. g) Flow cytometric analysis on the expression levels of M1 microglia ratio (F4/80/ CD86+). *n* = 6 for each group. Error bars denote mean ± SEM, ns, no significance, *p < 0.05, **p < 0.01, and ***p < 0.001.

First, microglia were treated with different concentrations of LPS. The expression of Sting and cGas peaked 24 h after treatment with 5 µg mL^−1^ LPS (**Figure** **3**a). Western blotting results showed that Sting and cGas expression and Irf3 and P65 phosphorylation levels were significantly increased in LPS‐induced microglia (Figure 3b). The expression of Sting increased in LPS‐induced microglia and decreased after treatment with C‐176 (Sting inhibitor) (Figure 3c; Figure S3a, Supporting Information). The expression of Sting and phosphorylation levels of P65 and Irf3 were also downregulated after treatment with C‐176 (Figure 3d). An immunofluorescence assay was performed to assess the effect of C‐176 on M1 polarization in LPS‐induced microglia. This blocking reduced iNOS expression (Figure 3e; Figure S3b, Supporting Information). The levels of IFN‐β, TNF‐α, and IL‐1β were significantly increased in LPS‐induced microglia. Moreover, the levels of these pro‐inflammatory cytokines were significantly decreased after treatment with C‐176 (Figure 3f). Flow cytometric analysis showed that C‐176 reduced the proportion of cluster of differentiation (CD86^+^) microglia (Figure 3g).

### 2.4 cGas–Sting Pathway Activation in LPS‐Induced Microglia Enhanced Neuron Death

**Figure 4:**
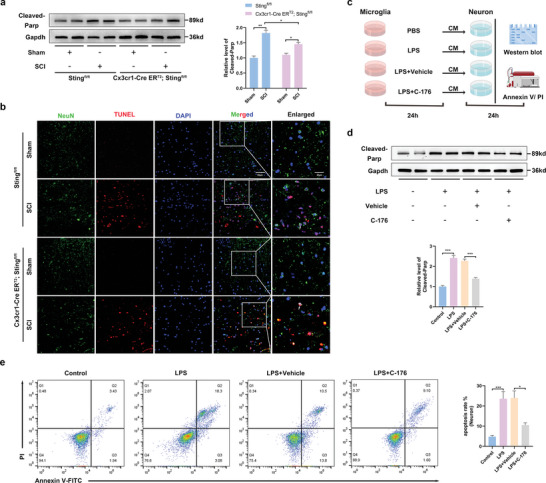
Sting signaling activation in LPS‐induced microglia enhanced neuron death. a) Western blot analysis of cleaved‐Parp expression in the perilesional tissues at 7 d after SCI. b) NeuN/TUNEL double immunostaining in the perilesional tissues 7 d after SCI (Scale bar: 50 µm). c) The protocol of in vitro experiments for detecting neurons (CATH.a) death regulated by Sting activation in primary microglia (by Figdraw). Primary microglia treated with PBS or LPS (5 µg mL^−1^) for 24 h. After removal of the supernatant, cells were cultured with DMEM culture medium for 24 h, and then the supernatant was collected as neurons conditioned medium for 24 h. d) Western blot analysis of cleaved‐Parp expression in neurons in each group. e) The neuron death rate was measured by Annexin V and PI staining. *n* = 6 for each group. Error bars denote mean ± SEM, ns, no significance, ^*^ *p* < 0.05, ^**^ *p* < 0.01, and ^***^ *p* < 0.001.

Neuronal destruction in SCI has been associated with pro‐inflammatory activation of microglia.^[^ ^4a^ ^]^ The role of Sting signaling in SCI was first analyzed in this study; Sting‐deficient mice were generated using cell‐specific knockout (KO) only in microglia. The level of cleaved poly‐ADP‐ribose polymerases (Parp) was significantly increased after SCI, which was reversed in the Cx3cr1‐Cre ER^T2^/Sting^fl/fl^ mice (**Figure** **4**a). Terminal deoxynucleotidyl transferase dUTP nick end labeling (TUNEL) staining showed that neuronal apoptosis was significantly reduced in Cx3cr1‐Cre ER^T2^/Sting^fl/fl^ mice after SCI (Figure 4b). To further confirm the effect of microglial activation on neurons, we performed in vitro experiments. Neurons were treated with different groups of microglia‐conditioned medium (CM) to verify the effects of microglial activation on neurons (Figure 4c). The level of cleaved Parp was increased significantly after LPS treatment, which was reversed by C‐176 treatment (Figure 4d). Flow cytometric analysis showed that neuronal death was lower in LPS‐ and C‐176‐treated microglial CM incubation than in LPS‐treated CM incubation (Figure 4e). Collectively, these results demonstrate that the activation of cGas–Sting pathway in microglia enhances neuronal death.

### 2.5 Reduced Expression of Mfn2 After SCI

**Figure 5:**
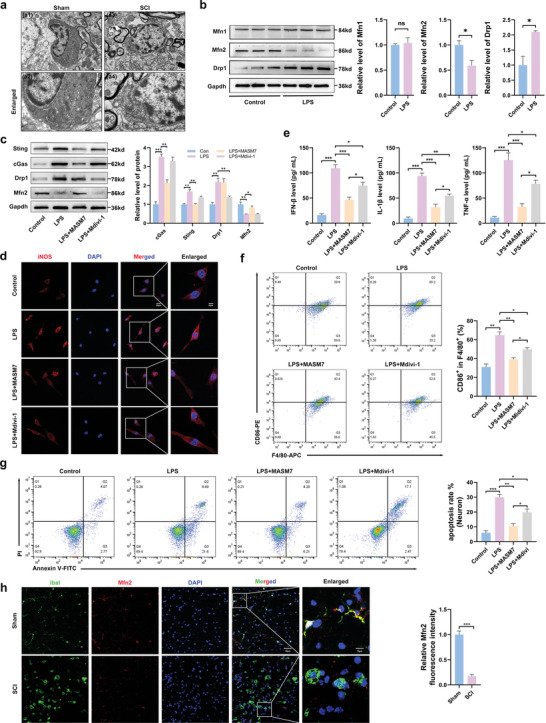
Mitochondria swelling and expression of mitofusin decreased after SCI. a) Representative ultrastructure of microglia in the perilesional tissues at 7 d after SCI (Scale bar: 1 µm (a1,a2) and 0.5 µm (a3,a4)). b) Western blot analysis of the Drp1, Mfn1, and Mfn2 levels in microglia of Control and LPS. c) Western blot analysis of cGas, Sting, Drp1, and Mfn2 levels in microglia of Control and LPS, LPS+MASM7, and LPS+Mdivi‐1. d) Representative of immunofluorescence staining of M1 state (iNOS) in microglia (Scale bar: 20 µm). e) Levels of pro‐inflammatory cytokines, including IL‐1β, IFN‐β, and TNF‐α in the medium of microglia. f) Flow cytometric analysis on the expression levels of M1 ratio (F4/80/ CD86+). g) The neuron death rate measured by Annexin V and PI staining. *n* = 6 for each group. h) Mfn2 /Iba1 double immunostaining in the perilesional tissues 7 d after SCI (Scale bar: 50 µm). Error bars denote mean ± SEM, ns, no significance, ^*^ *p* < 0.05, ^**^ *p* < 0.01, and ^***^ *p* < 0.001.

Ultrastructural changes in microglia after SCI were observed using transmission electron microscopy (TEM). The shape of the microglia in the sham group was normal, and most mitochondria were oval, with a highly folded inner membrane protruding inward to form cristae and a uniform outer membrane completely covering the organelles (**Figure** **5**a,a1,a2). In the SCI group, microglia showed mitochondrial swelling, destruction and disappearance of mitochondrial cristae, and mitochondrial membrane integrity (Figure 5a,a3,a4). These results suggest that damage to mitochondrial structure occurs after SCI. In mammals, mitochondrial fission is mediated by a single dynein‐related protein, Drp1, while mitochondrial fusion is primarily mediated by Mfn1 and Mfn2.^[^ ^16^ ^]^ In this study, we detected changes in the expression of these proteins in LPS‐induced microglia. Results showed that Drp1 levels increased and Mfn2 levels decreased in LPS‐induced microglia; however, Mfn1 levels did not exhibit a statistically significant difference (Figure 5b). To determine which protein played a more important role in Sting activation, we performed rescue experiments. Mitofusin Activator Small Molecule‐7 (MASM7) is a molecule activator of mitochondrial fusion via Mfn1 and Mfn2 and the mitochondrial membrane (Opa1).^[^ ^17^ ^]^ Mitochondrial division inhibitor‐1 (Mdivi‐1) is a chemical compound used to modulate the regulation of mitochondrial dynamics; specifically, Mdivi‐1 allows for the inhibition of Drp1 in mitochondrial fission.^[^ ^18^ ^]^ The results showed that MASM7 significantly reversed the increase in Sting levels in LPS‐induced microglia, whereas Mdivi‐1 downregulated Sting expression; however, the differences were not statistically significant (Figure 5c). We further investigated the effects of Drp1 and Mfn2 on microglial phenotypes. Immunofluorescence staining confirmed that MASM7 and Mdivi‐1 downregulated the expression of the M1 marker, iNOS, with a more pronounced effect observed with MASM7 (Figure 5d). Moreover, MASM7 induced a more significant reduction in the expression of pro‐inflammatory cytokines (IFN‐β, TNF‐α, and IL‐1β) (Figure 5e). Furthermore, treatment with MASM7 significantly reduced the proportion of CD86^+^ microglia (Figure 5f). Flow cytometry analysis showed that MASM7 was more effective at reducing neuronal death (Figure 5g). Using double immunostaining for Mfn2 and Iba1, we further confirmed the decreased expression of Mfn2 in the activated microglia of mice with SCI (Figure 5h). These results suggest that the downregulation of Mfn2 can lead to activation of the Sting signaling pathway.

### 2.6 Mfn2‐Dependent Mitochondrial Fusion Inducing mtDNA Release Mediates Activation of Sting Signaling in LPS‐Induced Microglia

**Figure 6:**
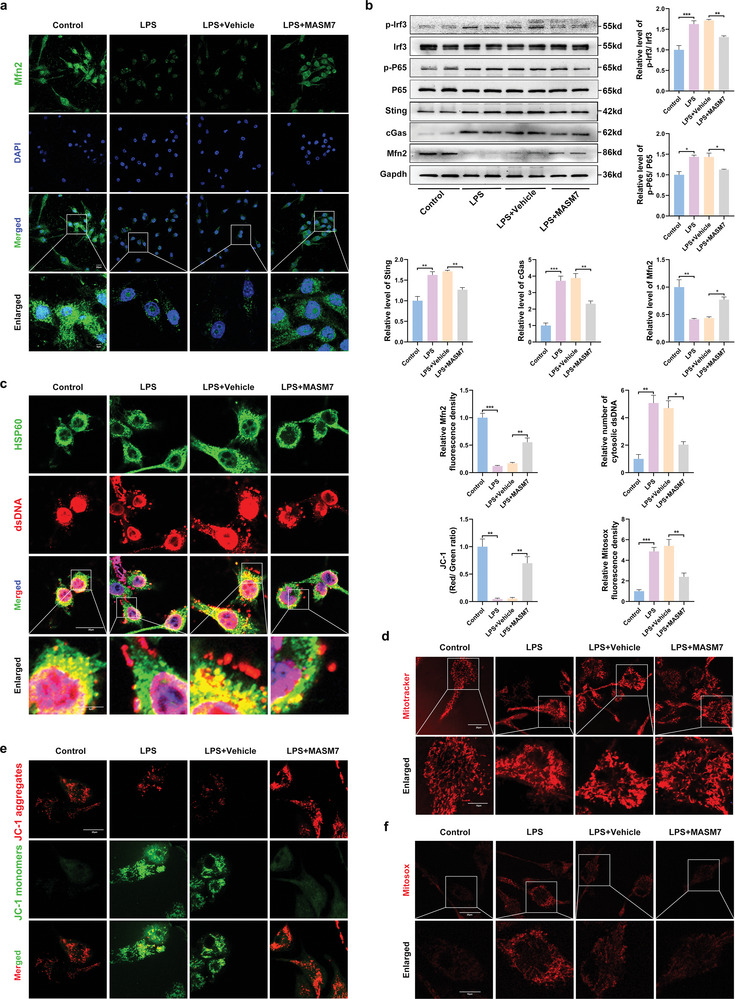
Mitochondrial fission‐induced mtDNA release mediates activation of Sting signaling in LPS‐induced microglial. a) Representative of immunofluorescence staining of Mfn2 in microglia in different groups (Scale bar = 20 µm). b) Western blot and quantitative analysis of Mfn2, cGas, Sting, Irf3, p‐Irf3, P65 and p‐P65 in microglia of Control, LPS, LPS + vehicle, and LPS + MASM7. c) dsDNA and HSP60 double immunostaining microglia in different groups (Scale bar: 20 µm). d) Representative MitoTracker fluorescence images illustrating mitochondrial morphology in microglia (Scale bar: 20 µm). e) Representative fluorescence staining of JC‐1 aggregates (red)/JC‐1 monomers (green) illustrating the MMP (Scale bar: 20 µm). f) Representative MitoSOX fluorescence images of mitochondria‐derived ROS (Scale bar: 20 µm). Error bars denote mean ± SEM, ns, no significance, ^*^ *p* < 0.05, ^**^ *p* < 0.01, and ^***^ *p* < 0.001.

**Scheme 1:**
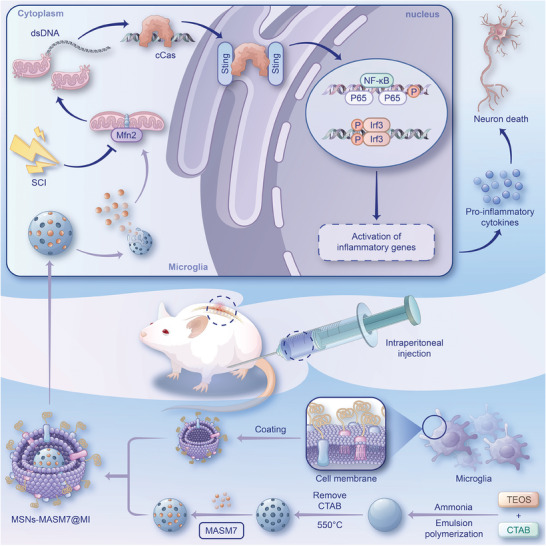
Schematic process of the activation of cGas‐Sting signaling mediated by Mfn2‐dependent release of mtDNA in microglia in SCI.

To further verify whether Mfn2 is an important target protein of mitochondrial morphological changes and functional damage caused by LPS, we chose the Mfn2 agonist, MASM7, for reverse intervention experiments. The immunofluorescence results showed that the expression of Mfn2 decreased in LPS‐induced microglia and increased after MASM7 treatment (**Figure** **6**a). We then detected the levels of Sting pathway‐related proteins using western blotting. We found that the levels of cGas, Sting, phosphorylated Irf3, and phosphorylated P65 were decreased after treatment with MASM7 (Figure 6b). Current evidence suggests that mtDNA can trigger the Sting pathway.^[^ ^19^ ^]^ We performed triple‐labeling of dsDNA, heat shock protein family D (HSP60) (a mitochondrial marker), and nuclei to assess whether mtDNA leaked into the microglial cytoplasm after LPS treatment. Immunofluorescence images showed that a large amount of mtDNA was released into the microglial cytoplasm after LPS treatment, which was reduced after MASM7 treatment (Figure 6c). After MASM7 treatment, the damage to mitochondrial morphology and function caused by LPS was significantly reduced, with reduced mitochondrial fragmentation (Figure 6d). Deep red staining for MitoSOX was used to determine mitochondrial reactive oxygen species (mtROS) levels, whereas matrix metalloproteinase (MMP) levels were measured using JC‐1 dye. LPS treatment significantly decreased MMP and increased ROS production, and these effects were alleviated by MASM7 treatment (Figure 6e,f). Taken together, these results suggest that abnormal Mfn2‐dependent mitochondrial fusion induces the release of mtDNA, which mediates the activation of the cGas‐Sting pathway (**Scheme** **1**).

### 2.7 MSNs‐MASM7@MI Efficiently Promoted Neuroprotection and Functional Recovery After SCI

Treatment of SCI is challenging because of the presence of the blood–spinal cord barrier.^[^ ^20^ ^]^ Previous studies have shown that nanoparticles coated with neutrophil and microglial membranes can pass through the blood–brain barrier,^[^ ^21^ ^]^ mediated by proteins expressed by these cells on the cell membrane.^[^^]^ To efficiently and accurately promote Mfn2 expression, we established a biomimetic nanodelivery platform for the targeted delivery of MASM7 into microglia. The preparation of membrane‐biomimetic MASM7@CM consisted of four steps: first, mesoporous silica nanoparticles (MSNs) were synthesized; second, MASM7 was adsorbed onto mesoporous channels of MSNs (termed MSNs‐MASM7); third, cell membranes were extracted using ultracentrifugation; and fourth, the pre‐extracted microglia, neurons, and astrocyte cell membranes were coated on the surface of the nanoparticles by extrusion to form MSNs‐MASM7@MI, MSNs‐MASM7@NE, or MSNs‐MASM7@AS (Scheme 1). The pore diameter of MSNs was mainly around 3 and 20 nm (Figure S4a, Supporting Information). The surface area and pore volume of MSNs were 35.7280 m^2^ g^−1^ and 0.117047 cm^3^ g^−1^, respectively (Figure S4b, Supporting Information). The pore size (adsorption average pore diameter) of MSNs was 12.6670 nm (Figure S4c, Supporting Information).

**Figure 7:**
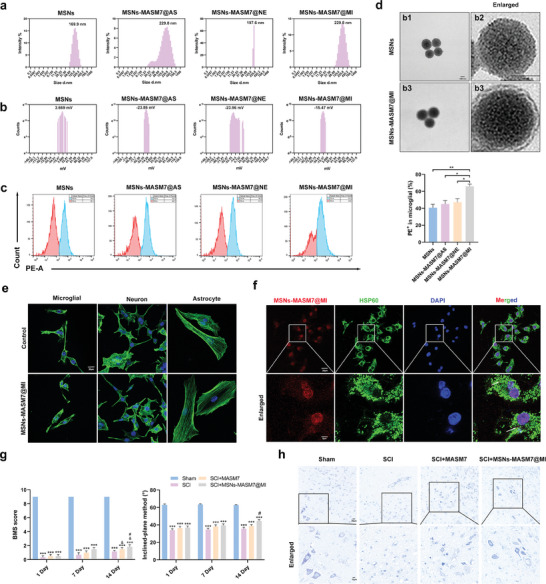
Nanomaterials carrying MASM7 promoted neuroprotection and functional recovery after SCI. a) The diameter distribution of MSNs, MSNs‐MASM7@AS, MSNs‐MASM7@NE, or MSNs‐MASM7@MI. b) The zeta potential distribution of MSNs, MSNs‐MASM7@AS, MSNs‐MASM7@NE, or MSNs‐MASM7@MI. c) Flow cytometric analysis of nanomaterials (coated by microglia membranes, neuron, and astrocyte membranes) uptake by primary microglial. d) TEM images of the MSNs and MSNs‐MASM7@MI (Scale bar = 50 nm). e) Fluorescence images of cellular morphology (F‐actin) in primary microglial, neuron, and astrocyte cells after 24 h (Scale bar = 20 µm). f) Fluorescence images of MSNs‐MASM7@MI and mitochondria (HSP60) in primary microglial after 24 h (Scale bar = 20 µm). g) Effects of different treatments on neurological function scores at 1, 7, and 14 d after SCI. h) Nissl staining in the perilesional tissues 7 d after SCI (Scale bar = 20 µm).

As shown in **Figure** **7**a, the size of MSNs was 169.9 nm, the MSNs‐MASM7@AS was 229.8 nm, the MSNs‐MASM7@NE was 197.6 nm, and the MSNs‐MASM7@MI was 229.8 nm. In addition, the surface of the MSNs became negatively charged after being coated with the cell membrane (Figure 7b). Flow cytometry results showed that microglial membranes (MSNs‐MASM7@MI) could enter microglia better compared to neurons (MSNs‐MASM7@NE) and astrocyte membranes (MSNs‐MASM7@AS) (Figure 7c). The TEM images of the MSNs and MSNs‐MASM7@MI nanoparticles are shown in Figure 7d. The size of MSNs‐MASM7@MI was 229.8–242 nm for 15 d and indicated good stability (Figure S4d, Supporting Information). The numerous pores provide space for loading MASM7 molecules.

In vitro, MSNs‐MASM7@MI showed no biological toxicity (Figure 7e) and delivered MASM7 to the mitochondria (Figure 7f). In vivo, MSNs‐MASM7@MI had no biological toxicity (Figure S4e, Supporting Information). In vivo, the mice treated with MSNs‐MASM7@MI had higher BMS and inclined‐plane method scores (Figure 7g). MSNs‐MASM7@MI‐treated mice showed a significant fluorescence signal from the spinal tissue (Figure S4f, Supporting Information), suggesting that MSNs‐MASM7@MI efficiently crossed the blood–spinal cord barrier. In addition, the number of Nissl‐stained positive cells in mice treated with MSNs‐MASM7@MI was significantly higher than that in the other groups (Figure 7h). Taken together, these data suggest that MSNs‐MASM7@MI can protect neurons and promote the recovery of neurological function in injured mice.

## 3 Discussion

SCI inflicts catastrophic physical and psychological trauma on patients and imposes a significant economic burden on the society owing to prolonged hospital stays, poor recovery outcomes, and increased reliance on nursing care.^[^ ^22^ ^]^ However, the mechanisms underlying the secondary injuries in SCI remain unclear. Microglia, the innate immune cells of the CNS, play a pivotal role in secondary SCI through excessive activation. In the current study, we established a link between hyperactive cGas‐Sting response and SCI. We demonstrated that the microglia‐specific KO of Sting attenuated the inflammatory response and promoted recovery from SCI. Mechanistically, we discovered that LPS‐induced activation of the cGas‐Sting pathway in microglia may be mediated through cytoplasmic mtDNA, which is released as a result of abnormal mitochondrial fusion caused by Mfn2 and the subsequent production of mtROS. Furthermore, the activation of Mfn2 by MASM7 significantly reduced the activation of Sting signaling in SCI. Collectively, our findings confirm that the Mfn2‐mtDNA‐cGas‐Sting axis is a potential target for SCI treatment. Additionally, we successfully developed a biomimetic microglial nanoparticle strategy for delivering MASM7 (named MSNs‐MASM7@MI), which showed promising results. These findings provide a new perspective on the occurrence of secondary SCI.

Sting is an innate immune adapter protein in the endoplasmic reticulum (ER) that functions synergistically with cGas to initiate an interferon‐based response to protect the host.^[^ ^7^ ^]^ The Sting pathway plays an important role in many CNS diseases.^[^ ^7^, ^23^ ^]^ However, little is known regarding the role of Sting in SCI. Our study found that Sting was significantly elevated after SCI. Moreover, by analyzing single‐cell data,^[^ ^14^ ^]^ we found that Sting was mainly expressed in microglia after SCI and was correlated with inflammation‐related pathways. Immunofluorescence analysis further confirmed these results. Consistent with these findings, a study examining the function of Sting in mice with subarachnoid hemorrhage concluded that Sting is mainly expressed in microglia.^[^ ^24^ ^]^ In addition, our IPA experiments validated that cGas‐Sting is an upstream molecule of these inflammation‐related molecules.

Microglia primarily originate in the embryonic yolk sac and populate the entire CNS during early neural development.^[^ ^25^ ^]^ Microglia can also regulate the innate and adaptive immune responses during pathological injury. The dysregulation of these responses is the basis for the pathogenesis of secondary SCI.^[^ ^26^ ^]^ It is widely acknowledged that microglia are the most dynamic cell population in the spinal cord after SCI.^[^ ^14^ ^]^ To further study the role of Sting in secondary SCI, we generated MKO mice. The results showed that Cx3cr1‐Cre ER^T2^/Sting^fl/fl^ mice exhibit better neurological function. Our western blot, TUNEL staining, and flow cytometry results showed that downregulating the expression of Sting could rescue the apoptosis of neurons. Interestingly, one study found that the Sting inhibitor C‐176 significantly attenuated neuroinflammation, thereby reducing neurological deficits in ischemic stroke mice.^[^ ^11b^ ^]^ Li et al.^[^ ^27^ ^]^ showed that activation of the cGas‐Sting pathway could produce microglial inflammasomes and pyroptosis in microglial cells, thereby amplifying the inflammatory response during brain ischemia/reperfusion injury. These results reveal that cGas‐Sting activation is the key to CNS injury. However, the role of Sting in the SCI model has not yet been fully understood. The cGas‐Sting pathway senses both microbial‐ and host‐derived dsDNA in the cytoplasm and initiates cellular innate immune responses in a Sting‐dependent manner.^[^ ^7^ ^]^ A potential source of cytosolic cGas‐agonistic self‐DNA is mtDNA.^[^ ^19^ ^]^ Our current study observed that microglia in the SCI group exhibited mitochondrial swelling, destruction, loss of mitochondrial cristae, and compromised mitochondrial membrane integrity. These findings suggest that Sting‐mediated neural cell death and inflammation may be attributed to mitochondrial dysfunction.

Mitochondria are the only organelles in cells that contain their own genome and are highly dynamic organelles that constantly undergo fission and fusion.^[^ ^28^ ^]^ Our results substantiate that Mfn2‐dependent abnormal mitochondrial fusion leads to Sting activation and suggest that MASM7 may be a potential drug for treating SCI. Mitochondrial dysfunction involves various processes, including mtDNA damage, depletion, and release.^[^ ^28^ ^]^ Leakage of mtDNA into the cytoplasm activates the cGas‐Sting immune pathway, which in turn, regulates innate immune responses and sterile inflammation.^[^ ^11^, ^19^ ^]^ In the current study, activation of the Sting pathway in LPS‐induced microglia was associated with the release of mtDNA into the cytoplasm. Meanwhile, upregulation of Mfn2 expression in microglia significantly reduced LPS‐induced mtDNA release. Mfn2 deficiency significantly reduced mitochondrial fusion, leading to mitochondrial fragmentation.^[^ ^29^ ^]^ Our results showed that MASM7 attenuated mitochondrial fragmentation in LPS‐induced microglia. Mitochondrial fragmentation has been reported to regulate mtROS production.^[^ ^30^ ^]^ In this study, we found that increased mtROS levels in LPS‐activated microglia were significantly downregulated by Mfn2 activation, suggesting that Mfn2‐mediated mitochondrial fusion regulates mtROS levels. Interestingly, it has been shown that the release of mtDNA in the cytoplasm is dependent on mtROS,^[^ ^31^ ^]^ and exogenous ROS (hydrogen peroxide) induces the release of mtDNA into the cytoplasm in mouse embryo fibroblasts.^[^ ^32^ ^]^ Based on these findings, it is probable that Mfn2 reduces mtDNA release by enhancing mitochondrial fragmentation and reducing mtROS production.

Nanomaterials possess significant potential for biomedical applications because of their ability to fine‐tune properties, such as loading capacity, drug protection, controlled drug release, and targeting capability.^[^ ^33^ ^]^ However, once inside the body, nanomaterials may absorb proteins and cellular components, leading to changes in their properties and recognition and elimination by the immune system.^[^ ^34^ ^]^ In recent years, functionalizing nanomaterials with cell membranes has emerged as a promising approach to endow nanomaterials with excellent biointerfacial properties.^[^ ^35^ ^]^ In the present study, we developed a microglial cell membrane‐coated silica nanoparticle drug delivery system that can deliver MASM7 to the site of SCI. This nanodelivery system was effective in improving dysfunction after SCI.

Taken together, our findings indicate that the activation of Sting signaling in microglia promotes neuronal death and inflammatory responses in SCI. Abnormal mitochondrial fusion mediated by Mfn2 promotes the generation of mtROS and the release of mtDNA into the cytoplasm. This mediates the activation of cGas‐Sting signaling pathway in microglia. In addition, we established a biomimetic microglial nanoparticle strategy to deliver MASM7 (named MSNs‐MASM7@MI) to improve dysfunction after SCI. These findings provide new evidence for the involvement of cGas‐Sting in the development of microglia‐mediated SCI and provide new therapeutic strategies for treating SCI.

## 4 Experimental Section

### Animals and Ethical Considerations

Male wild‐type (WT) C57BL/6 mice weighing 20–25 g and aged 8–12 weeks were obtained from the Animal Center of Fourth Military Medical University. MKO mice were generated by breeding Sting‐flox mice with Cx3cr1‐Cre ER^T2^ mice (Shanghai Model Organisms Center, Inc, Shanghai, China). All the mice were raised in a standard animal room under controlled environmental conditions. The indoor temperature was kept at 20–25 °C, the relative humidity of the air was kept at 40–70%, the number of air changes was 15 times h^−1^, and the alternating time between day and night was 12 h/12 h. During the rearing process, mice had free access to food and water. All experimental procedures were approved by the Ethics Committee and Institutional Review Board of the Fourth Military Medical University (Air Force Military Medical University). Animal experiments were conducted in accordance with the National Institute of Health Guide for the Care and Use of Laboratory Animals.

### SCI Model

The mice were anesthetized with 1% sodium pentobarbital (60 mg kg^−1^), followed by laminectomy of the vertebrae T9 to expose the spinal cord. In the spinal cord contusion model, an impactor weighing 10 g was dropped vertically from a height of 30 mm onto the surface of the exposed T9 spinal cord for 3 s. The mice were monitored daily to avoid infection and abnormal wound healing. Additionally, to assist the mice in voiding their bladders, they were manually stimulated by gentle squeezing once daily until they were euthanized.

### Behavioral Analysis

In this study, BMS was used to evaluate the hindlimb motor function of mice.^[^ ^36^ ^]^ The inclined‐plane method was used to indirectly evaluate trunk stability and hindlimb strength after SCI.^[^ ^37^ ^]^ To minimize any potential subjective bias in the scoring process, a double‐person and double‐blind approach was adopted.

### Analysis of Bulk RNA‐Seq and scRNA‐Seq from Spine Tissue

Bulk RNA‐seq and scRNA‐seq datasets were obtained from Figshare^[^ ^38^ ^]^ and analyzed using the R package. The thresholds for identifying DEGs were a *p*‐value < 0.05 and an absolute fold change ≥ 2. scRNA‐seq data (*n* = 1) were normalized and clustered using Seurat (version: v3.1.5). The top 2000 DEGs were selected for principal component analysis (PCA). The JackStraw function in Seurat was used to identify the optimal number of PCA components. Graph‐based algorithms in the PCA space and Uniformed Manifold Approximation and Projection (UMAP) dimensionality reduction techniques were employed for cell clustering and visualization. Pseudotime analysis and single‐cell trajectory analysis of microglia were performed using Monocle 2 (version: v14.0.80) in R. Dimensionality reduction was achieved using the DDRTree algorithm, and single‐cell trajectories were constructed using the orderCells function. Trajectories were visualized using the plot_cell_trajectory function. To identify the differential genes in the two microglial cell subpopulations, Gene Ontology (GO) and Kyoto Encyclopedia of Genes and Genomes (KEGG) databases were used for pathway enrichment analysis, with a resolution of 0.05. IPA was performed using QIAGEN's Ingenuity Pathway Analysis (IPA; QIAGEN Redwood Coty, www.qiagen.com/ingenuity).

### Cell Culture

Primary mouse microglia (CP‐M110) were purchased from Procell Life Science & Technology Co. Ltd. (Wuhan, China), which were cultured in a cell culture incubator with medium containing fetal bovine serum (FBS), growth supplements, and penicillin/streptomycin at a concentration of 5% CO_2_ at 37 °C. Mouse nerve cell lines (CATH.a) were grown in Dulbecco's modified Eagle medium (DMEM) (Gibco, USA) supplemented with 10% FBS (InCellGene, USA) and 1% penicillin‐streptomycin solution. All the cells were validated by the suppliers. Primary cultures of cortical astrocytes were prepared as described previously.^[^ ^39^ ^]^ Primary astrocytes were grown in DMEM/F12 (Gibco, USA) supplemented with 10% FBS (InCellGene, USA) and 1% penicillin‐streptomycin solution.

### Drug Administration

To inhibit Sting, microglia were treated with 1 µm C‐176 (MedChemExpress, USA).^[^ ^40^ ^]^ To inhibit Drp1, microglia were treated with 10 µm Mdivi‐1 (Selleck, USA).^[^ ^18^ ^]^ To activate Mfn2, microglia were treated with 1 µm MASM7 (MedChemExpress, USA).^[^ ^17^ ^]^ The same volume of PBS was used as the control. MASM7 (30 µg g^−1^) or vehicle (1% DMSO + corn oil) was administered intraperitoneally 30 min after SCI.

### Enzyme‐Linked Immunosorbent Assay (ELISA)

The mice were sacrificed before and after SCI and spinal cord samples were collected from the injury site. Cell culture supernatants containing secreted components were collected by transferring the medium to sterile tubes, followed by centrifugation at 2000–3000 rpm for 20 min. After centrifugation, the supernatant was collected. The collected supernatant samples were then analyzed using the respective ELISA kits (RK00420, RK00027, and RK00006, ABclonal) to measure IL‐1β, IFN‐β, and TNF‐α levels. All the ELISA tests were performed in accordance with the manufacturer's instructions (ABclonal, Wuhan, China).

### Nissl Staining

On day 7 after SCI, axial spinal cord sections of C57BL/6 mice were stained with formyl violet (for Nissl bodies). The Nissl‐positive cells in each visual field were counted for statistical analysis.

### Western Blotting

Proteins from trauma tissues, primary microglia, and neurons were prepared using ice‐cold radioimmunoprecipitation assay lysis buffer containing phosphatase and protease inhibitors. Protein samples (20 µg per lane) were separated by 10–12% sodium dodecyl sulphate‐polyacrylamide gel electrophoresis and transferred to polyvinylidene fluoride membranes (Millipore). The membranes were incubated at 4 °C overnight with the primary antibodies after blocking them with 5% nonfat milk. Primary antibodies were anti‐Sing (1:1000; 19851‐1‐AP, ProteinTech); anti‐cGas (1:1000; A8335, ABclonal); anti‐P65 (1:1000; 80979‐1‐RR, ProteinTech); anti‐p‐P65‐S536 (1:1000, #11 014, Signalway Antibody); anti‐Irf3 (1:1000, A2172, Abclonal), anti‐p‐Irf3‐S396 (1:1000, AP0623, Abclonal); anticleaved Parp (1:1000, #94 885, Cell Signaling Technology); anti‐Drp1 (1:1000; D6C7, Cell Signaling Technology); anti‐Mfn2 (1:1000, 12186‐1‐AP, ProteinTech); anti‐Mfn1 (1:1000, A9880, ABclonal); and antiglyceraldehyde 3‐phosphate dehydrogenase (Gapdh) (1:1000, 60004‐1‐Ig, ProteinTech). The membranes were then incubated with antimouse or antirabbit secondary antibodies (1:5000; AS003, AS014, ABclonal, Wuhan, China). Finally, the membranes were incubated with an enhanced chemiluminescence reagent (ABclonal, Wuhan, China).

### Immunofluorescence

Staining was performed using standard immunohistochemical procedures as previously described.^[^ ^41^ ^]^ Sections and primary microglia were washed 3 times with 1 × PBS and blocked with 5% normal goat serum (Gibco) and 0.2% Triton X‐100 for 1 h. Then, sections were incubated with specific antibodies at 4 °C overnight: anti‐Sting (1:200; 19851‐1‐AP, ProteinTech), anti‐Mfn2 (1:200; ab124773, Abcam), anti‐Iba1 (1:200; ab289874, Abcam), and anti‐iNOS (1:200; ab178945, Abcam). 4′,6‐diamidino‐2‐phenylindole (DAPI) (1:1000, Invitrogen) was used to label the nuclei. All sections and microglia were imaged using a Nikon confocal microscope and analyzed using ImageJ software.

### Flow Cytometry Analysis on the Activation Status of Primary Microglia

PE anti‐CD86 (ProteinTech, China) and APC anti‐F4/80 (BioLegend Co., USA) were used to detect the activation status of primary microglia in different groups. Different groups of primary microglia were washed with PBS and resuspended in the binding buffer. Then the primary microglia were stained with PE anti‐CD86 and APC anti‐F4/80 at 4 °C for 15 min in the dark. Flow cytometry (BD Biosciences) was used to immediately detect the samples, and FlowJo software (v10.6.2) was used to analyze the results.

### Flow Cytometric Analysis on the Apoptosis Levels of Neurons

Different groups of neurons were stained using annexin V/propidium iodide (PI) staining kits (ABclonal, China). Different groups of neurons were washed with PBS and resuspended in the binding buffer. The neurons were stained with annexin V‐fluorescein isothiocyanate (FITC) and PI for 15 min at 4 °C in the dark. Flow cytometry (BD Biosciences) was used to immediately detect the samples, and FlowJo software (v10.6.2) was used to analyze the results.

### TUNEL Staining

For NeuN and TUNEL costaining, cryosections were first stained with NeuN antibody (1:200; GB11138, Servicebio) overnight at 4 °C. TUNEL staining was used to detect apoptosis according to the manufacturer's protocol (Servicebio). The results are expressed as the number of double positives for live TUNEL and NeuN neurons in cells mm^−1^.^[^ ^42^ ^]^

### Analysis of Mitochondrial Morphology and Function

Mitochondria were analyzed for morphology and function as previously described.^[^ ^43^ ^]^ Primary microglia were seeded in confocal dishes and subjected to appropriate manipulations. A confocal microscope was used to analyze the mitochondrial morphology of the cells incubated for 30 min with 10 nm MitoTracker green (M7514, Life Technologies). MMP and mtROS production was assessed after 30 min of incubation with 10 nm JC‐1 (C2006, Beyotime) and 5 nm MitoSOX (M36008, Invitrogen). ImageJ software was used to quantify the relative fluorescence levels of cells captured under a confocal microscope.

### Synthesis of MSNs

MSNs were prepared as previously reported.^[^ ^44^ ^]^ The SiO_2_ nanoparticles were synthesized via three‐phase emulsion polymerization. Briefly, cetyltrimethylammonium bromide (CTAB, 0.1 g) was dissolved in a solution of n‐hexyl alcohol (8 mL), cyclohexane (37 mL), deionized water (2 mL), and Triton X‐100 (12 mL). After stirring for 0.5 h, tetraethoxysilane (TEOS, 0.5 mL), and ammonia water (0.5 mL) were added to the solution and stirred at 100 °C for another 24 h. After cooling, the mixture was separated by centrifugation at 12 000 rpm for 10 min and washed several times with ethanol and water. Then, the CTAB was removed at 550 °C for 6 h and the MSNs were obtained.

To encapsulate MASM7, the MSNs (10 mg) were added to 10 mL of MASM7 solutions (500 µg mL^−1^) and stirred for 12 h at room temperature. MSNs‐MASM7 were separated from the unincorporated MASM7 by centrifugation.

The cell membrane‐cloaked MSNs‐MASM7 nanoparticles (MSNs‐MASM7@CM) were obtained as follows (Scheme 1): Extraction of lipids from cells: The collected cells (primary microglia, neuron, and astrocyte) were treated with 10 mm Hepes buffer (0.25 m sucrose and 1 mm MgCl_2_) at pH 7.5, and then treated with ultrasound for 50 times, 30 s each time. The cell membrane (precipitate) was separated by centrifugation at 6000 rpm for 5 min. After mixing silica and the cell membrane equally, SiO_2_ coated with the cell membrane (MI, NE, and AS) was used to prepare nanoparticles using a hand‐pushed liposome extruder.

### Flow Cytometry Analysis on the Uptake of the Nanoparticle by Primary Microglia

The primary microglia were seeded into 6‐well plates at a density of 1 × 10^6^ units per well and incubated overnight; fresh culture media supplemented with MASM7@MI, MSNs‐MASM7@NE, or MSNs‐MASM7@AS were then added for another 30 h of incubation at 37 °C. Flow cytometry (BD Biosciences) was used to immediately detect the samples, and FlowJo software (v10.6.2) was used to analyze the results.

### Characterization of MSNs‐MASM7@MI

The morphologies of MSNs‐MASM7 and MSNs‐MASM7@MI were examined using TEM (JEM‐1230, Japan). For TEM imaging, the treated samples were dropped onto the surface of an ultrathin copper grid and then stained with a 1% phosphotungstate solution for 1 min. The hydrodynamic size of nanoparticle aggregates was characterized using a Zetasizer Nano‐ZS dynamic light scattering device. The zeta potentials of MSNs‐MASM7 and MSNs‐MASM7@MI were evaluated by dynamic light scattering (DLS) using the Delsa Nano C Particle analyzer (BECKMAN Coulter Instruments, USA).

### Statistical Analysis

Statistical analyses were performed by using GraphPad Prism (version 9.0). The continuous variables are presented as the means ± standard error of the mean (normal distribution) or medians with interquartile ranges (skewed distribution) from at least three independent experiments. Unpaired two‐tailed Student's *t*‐test was used to compare two independent groups, and multiple groups were compared using a one‐way analysis of variance. A two‐sided *p*‐value < 0.05 was considered statistically significant.

## Conflict of Interest

The authors declare no conflict of interest.

## Author Contributions

F.‐L.W., T.‐F.W., and C.‐L.W. contributed equally to this work. C.‐P.Z., F.‐L.W., J.‐X.Q., and C.‐L.W. conceived research ideas, designed experiments, analyzed data, and wrote the manuscript. F.‐L.W., T.‐F.W., and C.‐L.W. performed experiments. Z.‐P.Z., J.‐W.Z., W.H, Z.T., M.‐R.D., X.‐D.Y., and X.‐X.L. helped to complete the experiments. C.‐P.Z., J.‐X.Q., and Z.G. reviewed and edited the manuscript, discussed the results, and commented on the manuscript. All authors have read and approved the article.

## Supporting information

Supporting Information

## Data Availability

The data that support the findings of this study are available from the corresponding author upon reasonable request.

## References

[1] C. S. Ahuja , J. R. Wilson , S. Nori , M. R. N. Kotter , C. Druschel , A. Curt , M. G. Fehlings , Nat. Rev. Dis. Prim. 2017, 3, 17018. 28447605 10.1038/nrdp.2017.18

[2] M. B. Orr , J. C. Gensel , Neurotherapeutics 2018, 15, 541. 29717413 10.1007/s13311-018-0631-6 PMC6095779

[3] Z. Jie , C. J. Ko , H. Wang , X. Xie , Y. Li , M. Gu , L. Zhu , J. Y. Yang , T. Gao , W. Ru , S. Tang , X. Cheng , S. C. Sun , Sci. Adv. 2021, 7, 36. 10.1126/sciadv.abh0609 PMC8442891 34516909

[4] a) D. Samuel , K. Antje , Nat. Rev. Neurosci. 2011, 12, 388,;.21673720

[5] Z. Liu , X. Yao , W. Jiang , W. Li , S. Zhu , C. Liao , L. Zou , R. Ding , J. Chen , J. Neuroinflamm. 2020, 17, 90. 10.1186/s12974-020-01751-2 PMC7082940 32192500

[6] a) J. T. Qiao , C. Cui , L. Qing , L. S. Wang , T. Y. He , F. Yan , F. Q. Liu , Y. H. Shen , X. G. Hou , L. Chen , Metabol.: Clin. Exp. 2018, 81, 13. 10.1016/j.metabol.2017.09.010 29106945

[7] B. D. Paul , S. H. Snyder , V. A. Bohr , Trends Neurosci. 2021, 44, 83. 33187730 10.1016/j.tins.2020.10.008 PMC8662531

[8] S. Marchi , E. Guilbaud , S. W. G. Tait , T. Yamazaki , L. Galluzzi , Nat. Rev. Immunol. 2023, 23, 159. 35879417 10.1038/s41577-022-00760-x PMC9310369

[9] C. Zierhut , H. Funabiki , Trends Cell Biol. 2020, 30, 594. 32546434 10.1016/j.tcb.2020.05.006 PMC7368801

[10] S. Benmerzoug , B. Ryffel , D. Togbe , V. F. J. Quesniaux , Trends Immunol. 2019, 40, 719. 31262653 10.1016/j.it.2019.06.001

[11] a) K. P. Hopfner , V. Hornung , Nat. Rev. Mol. Cell Biol. 2020, 21, 501; 32424334 10.1038/s41580-020-0244-x

[12] Y. Liao , J. Cheng , X. Kong , S. Li , X. Li , M. Zhang , H. Zhang , T. Yang , Y. Dong , J. Li , Y. Xu , Z. Yuan , Theranostics 2020, 10, 9644. 32863951 10.7150/thno.47651 PMC7449914

[13] a) C. H. Yu , S. Davidson , C. R. Harapas , J. B. Hilton , M. J. Mlodzianoski , P. Laohamonthonkul , C. Louis , R. R. J. Low , J. Moecking , D. D. Nardo , K. R. Balka , D. J. Calleja , F. Moghaddas , E. Ni , C. A. McLean , A. L. Samson , S. Tyebji , C. J. Tonkin , C. R. Bye , B. J. Turner , G. Pepin , Cell 2020, 183, 636; 33031745 10.1016/j.cell.2020.09.020 PMC7599077

[14] C. Li , Z. Wu , L. Zhou , J. Shao , X. Hu , W. Xu , Y. Ren , X. Zhu , W. Ge , K. Zhang , J. Liu , R. Huang , J. Yu , D. Luo , X. Yang , W. Zhu , R. Zhu , C. Zheng , Y. E. Sun , L. Cheng , Signal Transduct. Target Ther. 2022, 7, 65. 35232960 10.1038/s41392-022-00885-4 PMC8888618

[15] B. Wasilewska , J. Najdzion , S. Szteyn , Folia Morphol. 2002, 61, 251. 12725492

[16] J. Nunnari , A. Suomalainen , Cell 2012, 148, 1145. 22424226 10.1016/j.cell.2012.02.035 PMC5381524

[17] E. Zacharioudakis , B. Agianian , V. Kumar Mv , N. Biris , T. P. Garner , I. Rabinovich‐Nikitin , A. T. Ouchida , V. Margulets , L. U. Nordstrøm , J. S. Riley , I. Dolgalev , Y. Chen , A. J. H. Wittig , R. Pekson , C. Mathew , P. Wei , A. Tsirigos , S. W. G. Tait , L. A. Kirshenbaum , R. N. Kitsis , E. Gavathiotis , Nat. Commun. 2022, 13, 3775. 35798717 10.1038/s41467-022-31324-1 PMC9262907

[18] Y. Yu , X. D. Peng , X. J. Qian , K. M. Zhang , X. Huang , Y. H. Chen , Y. T. Li , G. K. Feng , H. L. Zhang , X. L. Xu , S. Li , X. Li , J. Mai , Z. L. Li , Y. Huang , D. Yang , L. H. Zhou , Z. Y. Zhong , J. D. Li , R. Deng , X. F. Zhu , Signal Transduct. Target Ther. 2021, 6, 401. 34848680 10.1038/s41392-021-00790-2 PMC8632923

[19] A. P. West , W. Khoury‐Hanold , M. Staron , M. C. Tal , C. M. Pineda , S. M. Lang , M. Bestwick , B. A. Duguay , N. Raimundo , D. A. MacDuff , S. M. Kaech , J. R. Smiley , R. E. Means , A. Iwasaki , G. S. Shadel , Nature 2015, 520, 553. 25642965 10.1038/nature14156 PMC4409480

[20] T. I. Khan , S. Hemalatha , M. Waseem , Mol. Neurobiol. 2020, 57, 1978. 31900861 10.1007/s12035-019-01862-9

[21] a) L. Feng , C. Dou , Y. Xia , B. Li , M. Zhao , P. Yu , Y. Zheng , A. M. El‐Toni , N. F. Atta , A. Galal , Y. Cheng , X. Cai , Y. Wang , F. Zhang , ACS Nano 2021, 15, 2263; 33426885 10.1021/acsnano.0c07973

[22] S. A. Quadri , M. Farooqui , A. Ikram , A. Zafar , M. A. Khan , S. S. Suriya , C. F. Claus , B. Fiani , M. Rahman , A. Ramachandran , I. I. T. Armstrong , M. A. Taqi , M. M. Mortazavi , Neurosurg. Rev. 2020, 43, 425. 29998371 10.1007/s10143-018-1008-3

[23] C.‐H. Yu , S. Davidson , C. R. Harapas , J. B. Hilton , M. J. Mlodzianoski , P. Laohamonthonkul , C. Louis , R. R. J. Low , J. Moecking , D. De Nardo , K. R. Balka , D. J. Calleja , F. Moghaddas , E. Ni , C. A. McLean , A. L. Samson , S. Tyebji , C. J. Tonkin , C. R. Bye , B. J. Turner , G. Pepin , M. P. Gantier , K. L. Rogers , K. McArthur , P. J. Crouch , S. L. Masters , Cell 2020, 183, 636. 33031745 10.1016/j.cell.2020.09.020 PMC7599077

[24] Y. Peng , J. Zhuang , G. Ying , H. Zeng , H. Zhou , Y. Cao , H. Chen , C. Xu , X. Fu , H. Xu , J. Li , S. Cao , J. Chen , C. Gu , F. Yan , G. Chen , J. Neuroinflammation. 2020, 17, 165. 32450897 10.1186/s12974-020-01830-4 PMC7247752

[25] Y. N. Jassam , S. Izzy , M. Whalen , D. B. McGavern , J. El Khoury , Neuron 2017, 95, 1246. 28910616 10.1016/j.neuron.2017.07.010 PMC5678753

[26] S. Brockie , J. Hong , M. G. Fehlings , Int. J. Mol. Sci. 2021, 9706, 22. 10.3390/ijms22189706 PMC8470129 34575871

[27] Q. Li , Y. Cao , C. Dang , B. Han , R. Han , H. Ma , J. Hao , L. Wang , EMBO Mol. Med. 2020, 12, 11002. 10.15252/emmm.201911002 PMC7136961 32239625

[28] M. Giacomello , A. Pyakurel , C. Glytsou , L. Scorrano , Nat. Rev. Mol. Cell Biol. 2020, 21, 204. 32071438 10.1038/s41580-020-0210-7

[29] G. Meng , J. Liu , S. Liu , Q. Song , L. Liu , L. Xie , Y. Han , Y. Ji , Br. J. Pharmacol. 2018, 175, 1126. 28503736 10.1111/bph.13861 PMC5866985

[30] C. Duan , L. Wang , J. Zhang , X. Xiang , Y. Wu , Z. Zhang , Q. Li , K. Tian , M. Xue , L. Liu , T. Li , Redox Biol. 2020, 37, 101706. 32911435 10.1016/j.redox.2020.101706 PMC7490562

[31] K. Nakahira , J. A. Haspel , V. A. K. Rathinam , S.‐J. Lee , T. Dolinay , H. C. Lam , J. A. Englert , M. Rabinovitch , M. Cernadas , H. P. Kim , K. A. Fitzgerald , S. W. Ryter , A. M. K. Choi , Nat. Immunol. 2011, 12, 222. 21151103 10.1038/ni.1980 PMC3079381

[32] J. Kim , R. Gupta , L. P. Blanco , S. Yang , A. Shteinfer‐Kuzmine , K. Wang , J. Zhu , H. E. Yoon , X. Wang , M. Kerkhofs , H. Kang , A. L. Brown , S.‐J. Park , X. Xu , E. Zandee van Rilland , M. K. Kim , J. I. Cohen , M. J. Kaplan , V. Shoshan‐Barmatz , J. H. Chung , Science 2019, 366, 1531. 31857488 10.1126/science.aav4011 PMC8325171

[33] Z. Luo , Y. Lu , Y. Shi , M. Jiang , X. Shan , X. Li , J. Zhang , B. Qin , X. Liu , X. Guo , J. Huang , Y. Liu , S. Wang , Q. Li , L. Luo , J. You , Nat. Nanotechnol. 2023, 18, 647. 37081080 10.1038/s41565-023-01374-7

[34] S. M. Moghimi , A. C. Hunter , T. L. Andresen , Ann. Rev. Pharmacol. Toxicol. 2012, 52, 481. 22035254 10.1146/annurev-pharmtox-010611-134623

[35] R. Yuan , M. Liu , Y. Cheng , F. Yan , X. Zhu , S. Zhou , M. Dong , ACS Nano 2023, 17, 8204. 37071566 10.1021/acsnano.2c12190

[36] D. M. Basso , L. C. Fisher , A. J. Anderson , L. B. Jakeman , D. M. McTigue , P. G. Popovich , J. Neurotrauma 2006, 23, 635. 16689667 10.1089/neu.2006.23.635

[37] A. S. Rivlin , C. H. Tator , J. Neurosurg. 1977, 47, 577. 903810 10.3171/jns.1977.47.4.0577

[38] C. Li , Z. Wu , L. Zhou , J. Shao , X. Hu , W. Xu , Y. Ren , X. Zhu , W. Ge , K. Zhang , J. Liu , R. Huang , J. Yu , D. Luo , X. Yang , W. Zhu , R. Zhu , C. Zheng , Y. E. Sun , L. Cheng , figshare. Dataset, 2022, 10.6084/m9.figshare.17702045.v2.

[39] W. Shao , S.‐z. Zhang , M. Tang , X.‐h. Zhang , Z. Zhou , Y.‐q. Yin , Q.‐b. Zhou , Y.‐y. Huang , Y.‐j. Liu , E. Wawrousek , T. Chen , S.‐b. Li , M. Xu , J.‐n. Zhou , G. Hu , J.‐w. Zhou , Nature 2013, 494, 90. 23242137 10.1038/nature11748

[40] X. Bi , C. Du , X. Wang , X.‐Y. Wang , W. Han , Y. Wang , Y. Qiao , Y. Zhu , L. Ran , Y. Liu , J. Xiong , Y. Huang , M. Liu , C. Liu , C. Zeng , J. Wang , K. Yang , J. Zhao , Adv. Sci. 2021, 8, 2002738. 10.1002/advs.202002738 PMC7927614 33717842

[41] W. Cui , X. Wu , Y. Shi , W. Guo , J. Luo , H. Liu , L. Zheng , Y. Du , P. Wang , Q. Wang , D. Feng , S. Ge , Y. Qu , Cell Prolif. 2021, 54, e12964. 33314534 10.1111/cpr.12964 PMC7848954

[42] G. J. Hankey , Lancet 2017, 389, 641. 27637676 10.1016/S0140-6736(16)30962-X

[43] C. Gui , Y. Ren , J. Chen , X. Wu , K. Mao , H. Li , H. Yu , F. Zou , W. Li , Toxicol. Appl. Pharmacol. 2020, 388, 114874. 31881179 10.1016/j.taap.2019.114874

[44] X. He , D. Wang , P. Chen , Y. Qiao , T. Yang , Z. Yu , C. Wang , H. Wu , Chem. Commun. 2020, 56, 4785. 10.1039/d0cc00600a 32227029

