# Supplementary material for: Cytoplasmic Escape of Mitochondrial DNA Mediated by Mfn2 Downregulation Promotes Microglial Activation via cGas‐Sting Axis in Spinal Cord Injury
Source: Adv Sci (Weinh). 2023 Nov 27;11(4):2305442. doi: 10.1002/advs.202305442 (PMC10811505; doi:10.1002/advs.202305442)

ADVANCED SCIENCE
Open Access

Supporting Information

for *Adv. Sci.*, DOI 10.1002/advs.202305442

Cytoplasmic Escape of Mitochondrial DNA Mediated by Mfn2 Downregulation Promotes Microglial Activation via cGas-Sting Axis in Spinal Cord Injury

Fei-Long Wei, Tian-Fu Wang, Chao-Li Wang, Zhen-Peng Zhang, Jing-Wei Zhao, Wei Heng, Zhen Tang, Ming-Rui Du, Xiao-Dong Yan, Xiao-Xiang Li, Zheng Guo, Ji-Xian Qian* and Cheng-Pei Zhou**

Supplementary Figure 1. Cluster 1 microglia was a pro-inflammatory cluster.

a UMAP plot showing seven microglia clusters (subtypes). **b** GO enrichment analysis showing hallmark pathways associated with the differentially expressed genes upregulated in cluster 0 microglia compared to cluster 1 microglia samples. **c** KEGG enrichment analysis showing hallmark pathways associated with the differentially expressed genes upregulated in cluster 0 microglia compared to cluster 1 microglia samples.

Supplementary Figure 1

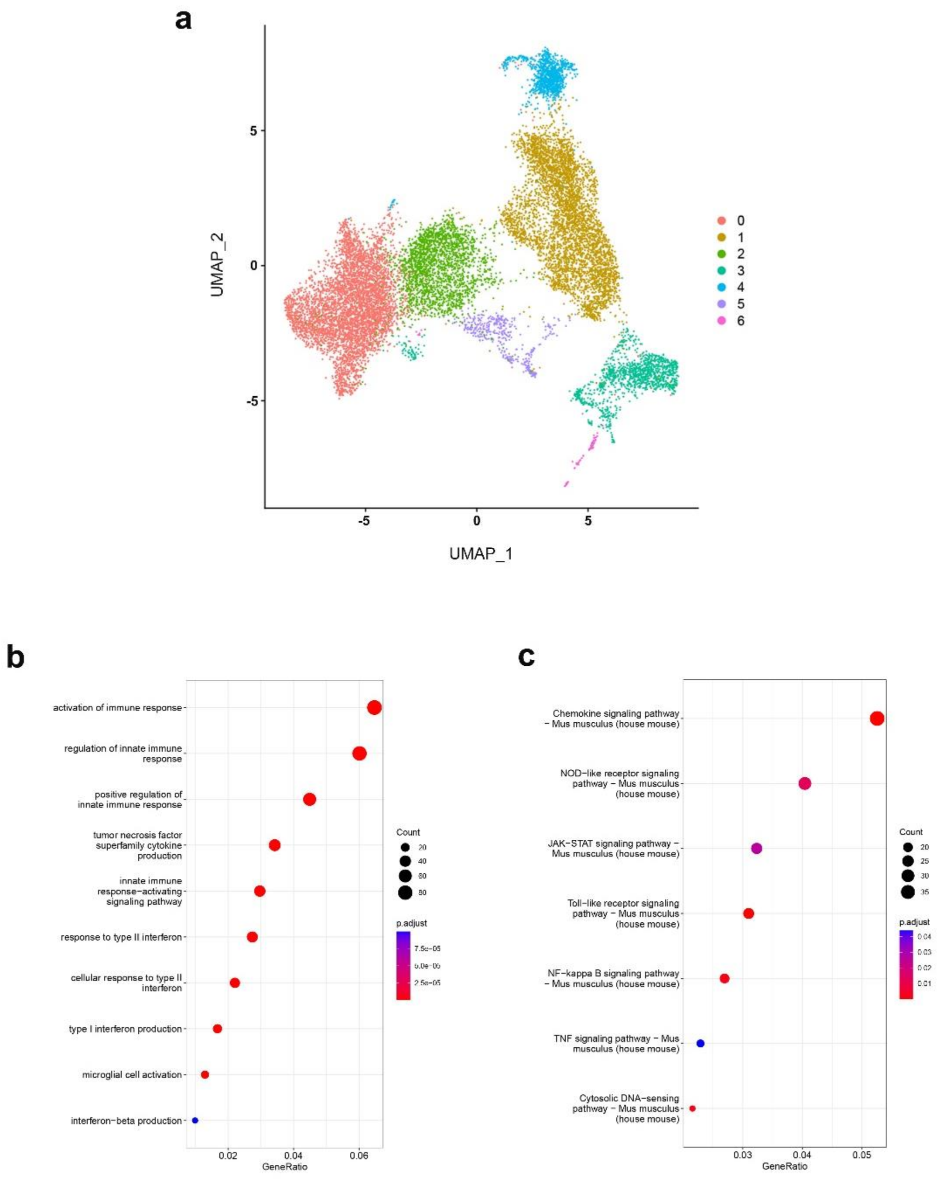

Supplementary Figure 2. a Number of positive Nissl staining cells. **b** Relative iNOS fluorescence density of microglia.

Supplementary Figure 2

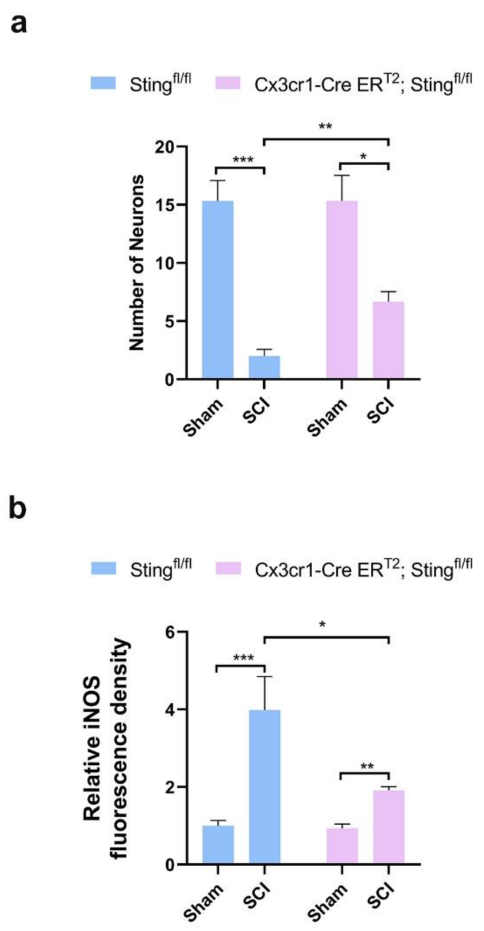

Supplementary Figure 3. a Relative Sting fluorescence density of microglia. **b** Relative iNOS fluorescence density of microglia.

Supplementary Figure 3

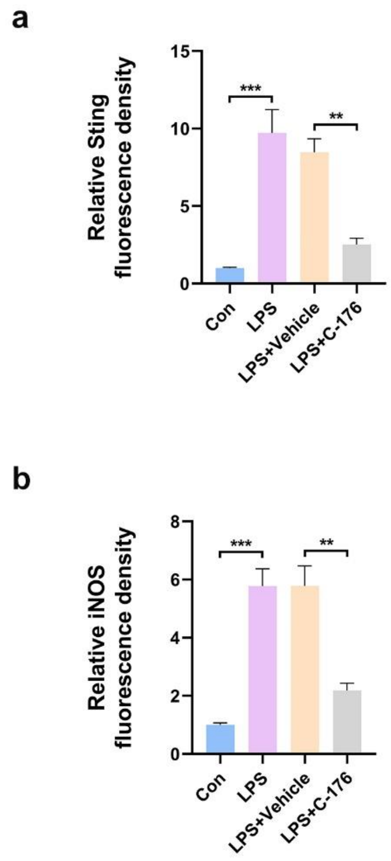

Supplementary Figure 4. a The pore volume of silica nanoparticles. **b** The surface area and pore volume of MSNs. **c** The pore size (adsorption average pore diameter) of SiO_2 nanoparticles. **d** the stability of MSNs-MASM7@MI. **e** Hematoxylin and eosin staining and histological analysis of main organs from MSNs-MASM7@MI-treated mice. **f** Fluorescence signals observed in the brain and spine tissue of mouse 6 h after intraperitoneal injection of MSNs-MASM7@MI.

Supplementary Figure 4

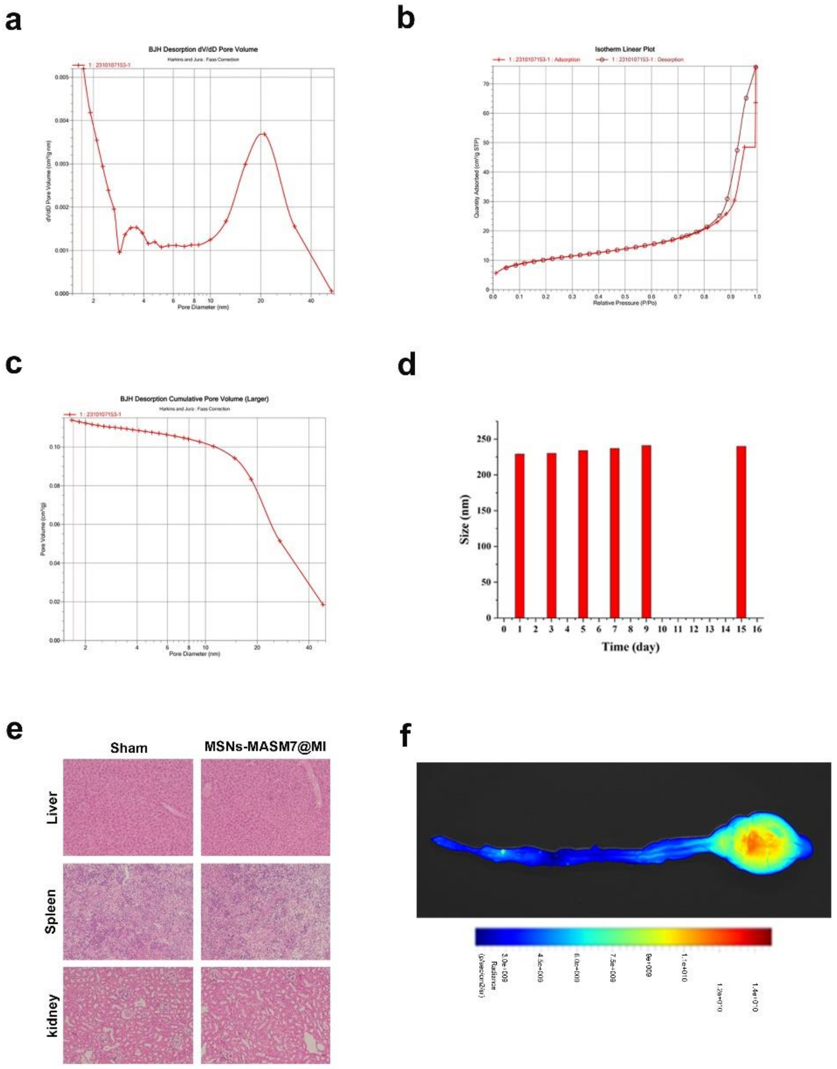

Supplement: Supplementary file 1 — Supporting Information [file ADVS-11-2305442-s001.pdf]
